# Supplementary material for: High COVID-19 transmission potential associated with re-opening universities can be mitigated with layered interventions
Source: Nat Commun. 2021 Aug 17;12:5017. doi: 10.1038/s41467-021-25169-3 (PMC8371131; doi:10.1038/s41467-021-25169-3)
Supplement: Supplementary file 1 — Supplementary Information [file 41467_2021_25169_MOESM1_ESM.pdf]

Supplementary information for
High COVID-19 transmission potential associated with re-opening universities can be mitigated with layered interventions.

Ellen Brooks-Pollock, Hannah Christensen, Adam Trickey, Gibran Hemani, Emily Nixon, Amy C. Thomas, Katy Turner, Adam Finn, Matt Hickman, Caroline Relton, Leon Danon

Supplementary Note 1. Data processing

The University of Bristol provided pseudonymised data relating to the academic year 2019/2020. The study complied with the University data protection policy for research studies (http://www.bristol.ac.uk/media-library/sites/secretary/documents/information-governance/data-protection-policy.pdf). We used this information to build a matrix of contacts between courses and year of study. In Figure S1 we show the schematic of how the contact matrix is built. Each line in the Raw data represents an individual student, and the columns show the relevant data fields (School name, Year, Term time postcode). This is transformed into a wide format matrix, W, where students that share accommodation, as indicated by a shared postcode, are summed up. In the final step, the wide format matrix, W, is multiplied by its transpose, W^T, and then normalised to give a household contact matrix h_{ij}. The entries of the H matrix denote the average number of contacts that a student in particular school has with any other school, through their accommodation (Supplementary figure 1).

Raw data

School name	Year	Term time postcode
Law	1	p1
Law	1	p2
Law	1	p2
Medicine	1	p1
Medicine	1	p1
Medicine	1	p1
Medicine	1	p2
Medicine	1	p3

Wide format

	p1	p2	p3
Law.1	1	2	0
Medicine.1	3	1	1

Contact matrix

	Law.1	Medicine.1
Law.1	0.67	1.67
Medicine.1	1	1.2

Supplementary figure 1: Stages involved in data process.

Supplementary Note 2. Living circles

We assumed that students could transmit infection to other individuals in their living circles. Living circles were defined as other students living at the same postcode, or where the number of students at the same postcode was greater than the maximum living circle size (24 in the baseline case), we randomly allocated students at that postcode to a living circle. Supplementary figure 2 shows the number of students living at a single postcode and whether a halls of residence was located at that same postcode. The average number of students living at a single postcode for private accommodation was 3.25, and for halls of residence was 137. 3.5% of postcodes were associated with more than 24 students; these could have been halls of residence or private houses near each other.

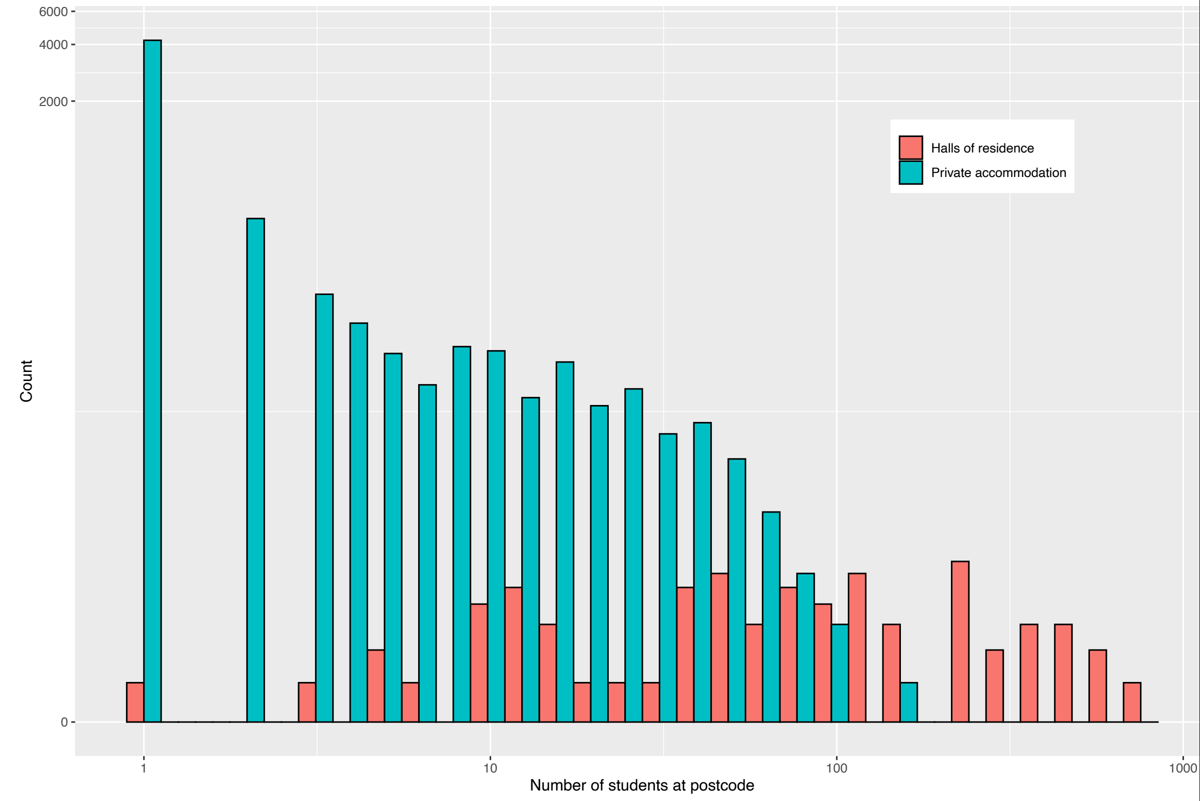

Supplementary figure 2: The number of students living at a single postcode from University of Bristol data for 2019/2020, and whether a halls of residence was located at that same postcode.

Supplementary Note 3. Estimating the reproduction number in a university setting.

In the university age population, we estimate that individuals have on average 10% more contacts than an individual in the general population using data from the Social Contact Survey. We also estimate that 25% of the cases in this population will show COVID-19 symptoms[1]. However, there is substantial uncertainty around the relative infectiousness of asymptomatic cases. In Supplementary figure 3 we vary the relative infectiousness of asymptomatic individuals under the assumption that they are less infectious than symptomatic individuals and assess the impact on R in the university R_U. Beginning with the reproduction number in the general population, R = 2.7, we observe that in the University population, R_U varies between 1.7 when ε=0.1 (due to the reduced infectiousness of asymptomatic cases), and 3.4 when ε=1 (due to the increased number of contacts in the university population).

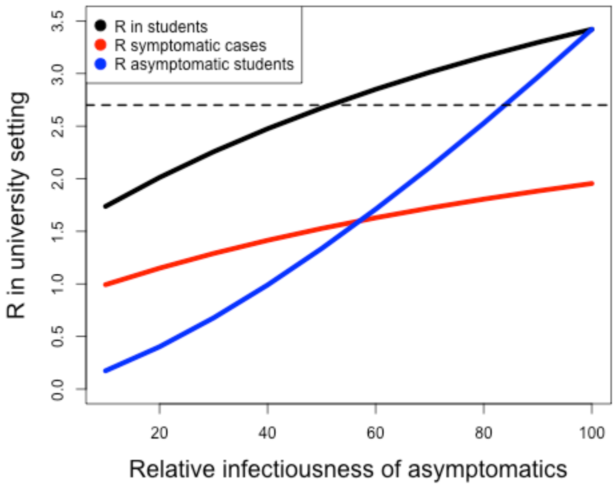

Supplementary figure 3: The reproduction number in a university setting as a function of the relative infectiousness of asymptomatic cases, under the assumption that $R = 2.7$ in the general population.

Supplementary Note 4. Sensitivity to model parameters and impact of asymptomatic transmission on model dynamics

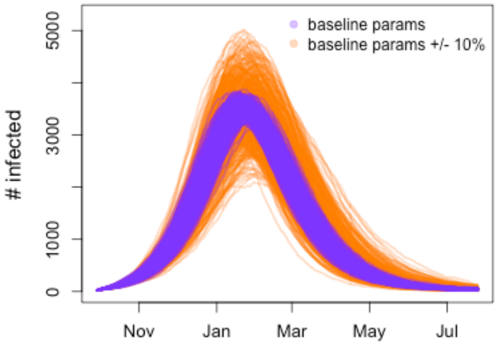

Supplementary figure 4: Epidemic trajectories for the model with baseline parameters and varying baseline parameters by +/- 10%.

Supplementary figure 4 shows the model sensitivity to parameter variation. Because the relative infectiousness of asymptomatic cases, the parameter ε, was the most uncertain and a key parameter for this population, we performed extensive simulations to analyse the sensitivity of our model to this parameter (Supplementary figure 5). The results are summarised in Figure S4 below. For low values ε (0.1 - 0.2), the epidemic peak is small and the epidemic continues at a low level throughout the academic year with the expected number of symptomatic cases barely rising above 1. For low-intermediate values of ε (0.3-0.5) the peak number of cases rises rapidly, and the epidemic increases in speed, peaking after the Christmas break. For high values of ε (0.6 and above) the epidemic peak is at or just before the Christmas break in the baseline case. The highest number of cases at Christmas is observed for $\varepsilon = 0.7$ in the baseline case. For $\varepsilon = 1$, a peak at Christmas is observed when mitigation strategies are implemented.

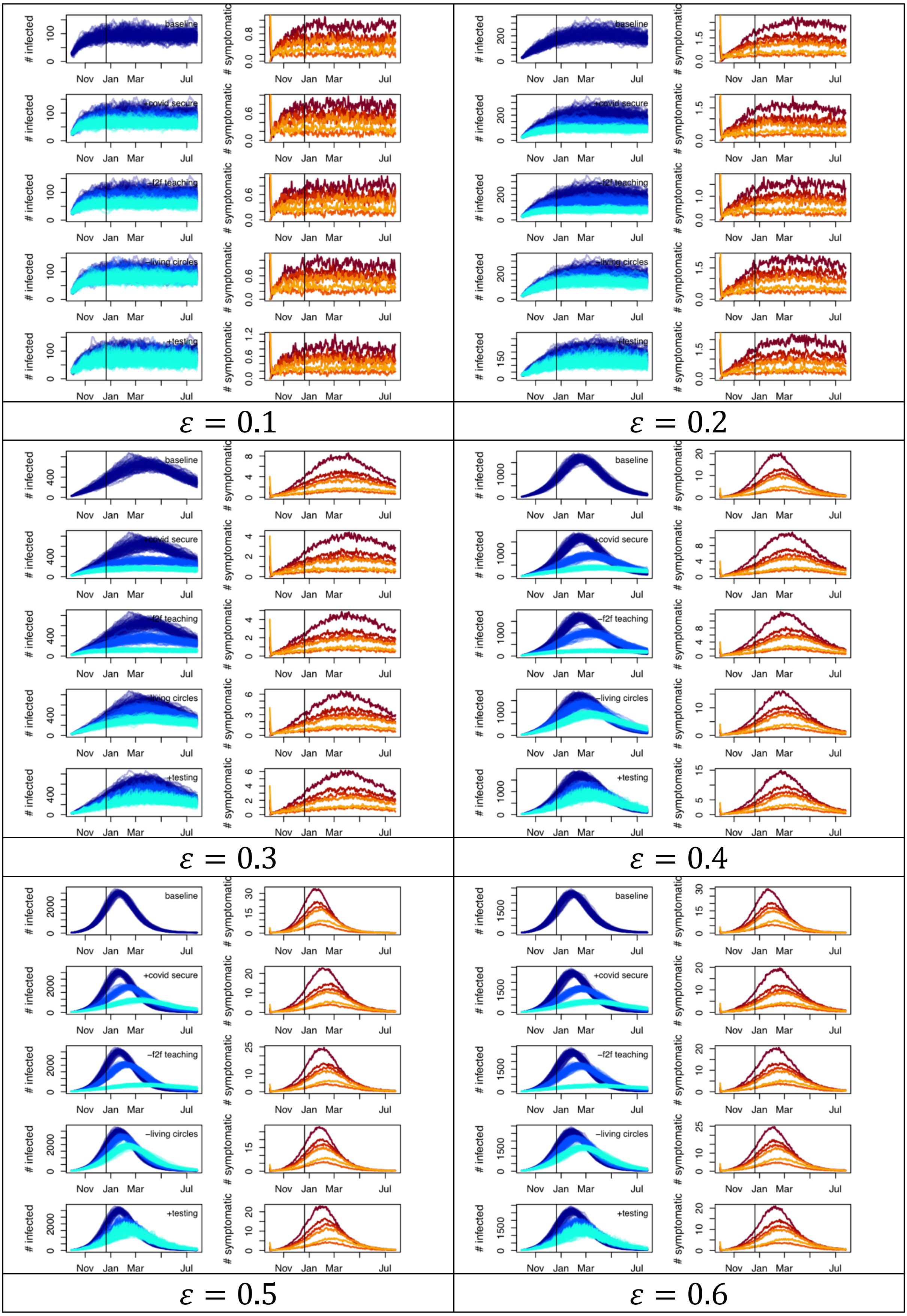

infected
symptomatic
baseline
+covid secure
−f2f teaching
−living circles
+testing
Nov
Jan
Mar
Jul
ε = 0.1
ε = 0.2
ε = 0.3
ε = 0.4
ε = 0.5
ε = 0.6

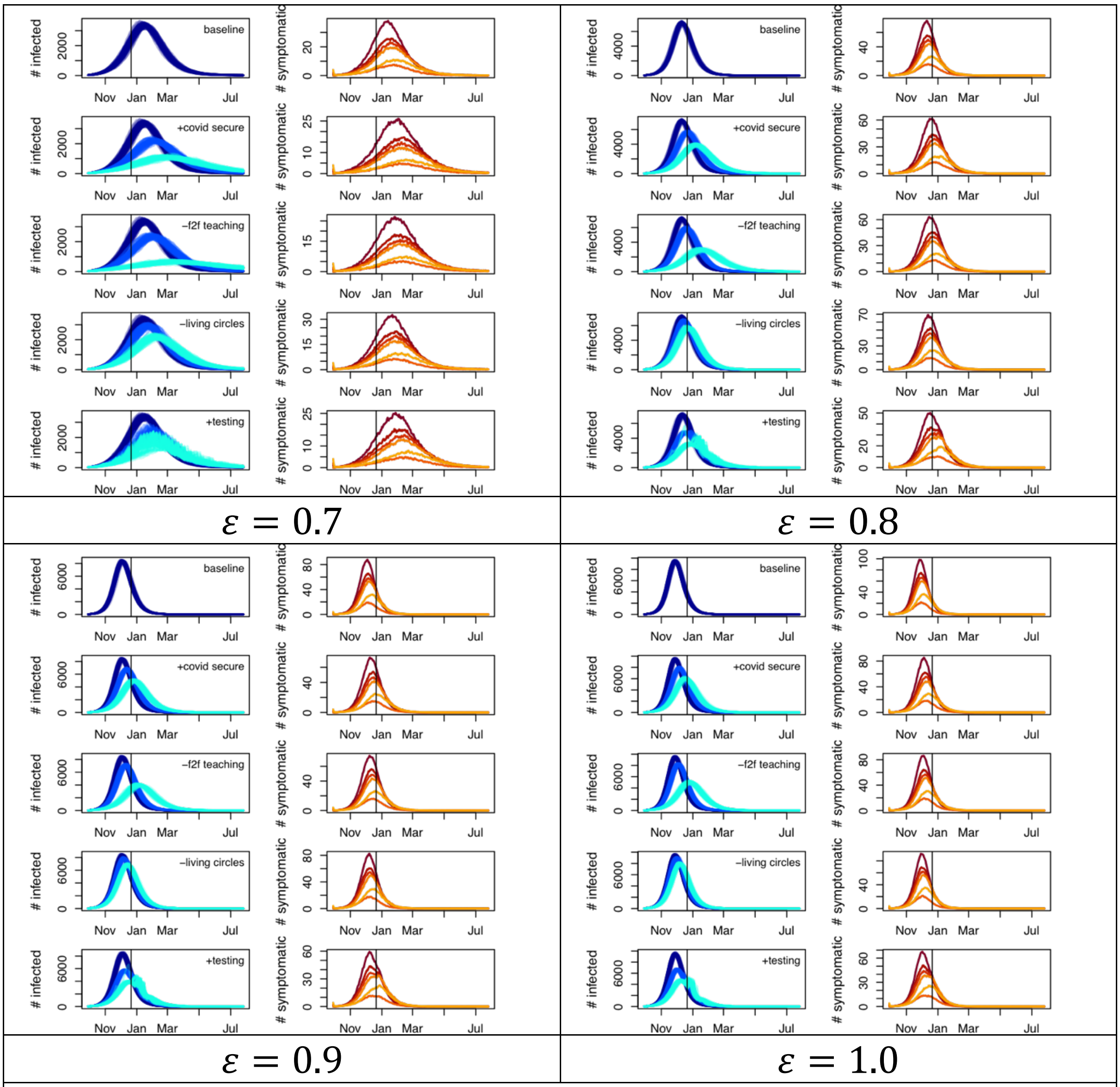

Supplementary figure 5: Epidemic trajectories for the baseline model and four types of interventions: implementing COVID security (25% and 50%), reduced face-to-face teaching (teaching contacts up to 15 and 5 students), smaller living circles (up to 20 and 14 students per living circle) and mass testing of all students (every 7 and 2 days). The figures show the impact of increasing the realtive infectiousness of asymptomatic cases (ε) and hence the reproduction number. Right panels: number of infected cases over time. Dark blue is the baseline model, royal blue is the first intervention, cyan is the second intervention; Left panels: Number of symptomatic cases by year group, with darker colours

Supplementary Note 5. Impact of testing frequency

To assess the robustness of our results, we performed sensitivity analysis of the relative asymptomatic infectiousness, as captured by ε on the results of impact of testing frequency (Supplementary table 1). We considered three values of ε, shown below in Supplementary figure 6.

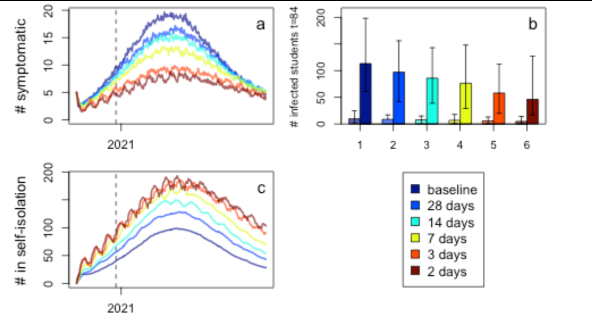

$\varepsilon = 0.3$

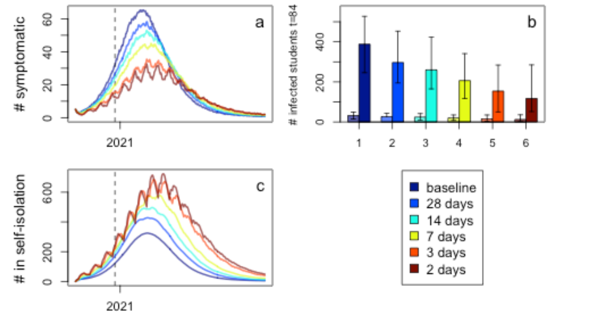

$\varepsilon = 0.5$

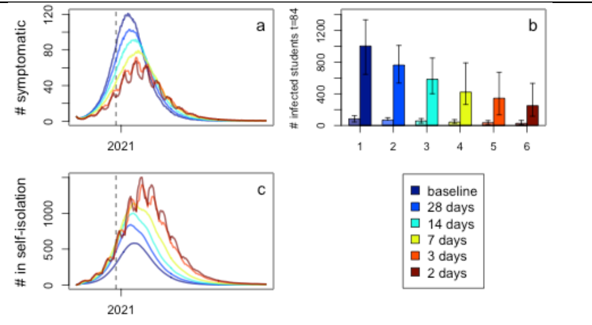

$\varepsilon = 0.7$

Supplementary figure 6: The impact of testing frequency on a) number of symptomatic cases; b) number of symptomatic and asymptomatic cases at the end of the first term and c) the number of students self-isolating for different values of asymptomatic infectiousness, $\varepsilon = 0.3, 0.5, 0.7$.

SCENARIO NUM	INTERVENTION	EPSILON	Ru	# symp cases at xmas (mean)	min	max	rank based on # symp cases	# asymp cases at xmas (mean)	min	max	R_xmas (%)	min	max	RANK RX	growthrate	min	max	doubling time (days)	min	max	R_end (%)	min	max
1	baseline	30	2.25	13	4	23	9	150	100	230	4.8	3.4	6.7	9	0.053	0.022	0.089	13	7.8	32	34	29	38
2	CS25	30	2.25	7.5	2	17	4	95	52	140	3.3	2.4	4.4	3	0.046	0.022	0.077	15	9	32	19	15	23
3	CS50	30	2.25	4.8	0	10	2	61	38	100	2.4	1.9	3.3	2	0.041		0.078	17	8.9		11	9.5	13
4	f2ft15	30	2.25	8.4	2	16	6	100	73	150	3.5	2.7	4.6	5	0.047	0.009	0.068	15	10	78	21	17	25
5	f2ft5	30	2.25	3.9	0	11	1	50	31	73	2.1	1.7	2.6	1	0.037		0.071	19	9.8		8.5	7.5	9.7
6	LC20	30	2.25	10	2	22	8	130	79	180	4.1	3	5.5	8	0.053	0.023	0.088	13	7.9	30	27	22	32
7	LC14	30	2.25	8	2	21	5	98	50	160	3.4	2.6	4.6	4	0.047	0.017	0.081	15	8.6	41	20	16	24
8	T7	30	2.25	9.8	1	20	7	98	47	190	4.1	2.6	6.1	7	0.025		0.072	28	9.6		28	23	34
9	T2	30	2.25	6.4	1	27	3	66	25	140	3.7	2.7	4.9	6			0.086		8.1		24	20	28
1	baseline	50	2.67	71	48	100	9	820	590	1000	19	13	24	9	0.073	0.028	0.1	9.5	6.9	25	73	71	75
2	CS25	50	2.67	35	15	53	5	410	270	550	10	7.4	13	5	0.065	0.04	0.091	11	7.6	17	60	58	63
3	CS50	50	2.67	16	3	36	2	200	120	300	5.7	4	7.7	2	0.057	0.029	0.086	12	8.1	24	41	36	44
4	f2ft15	50	2.67	39	19	60	7	460	320	620	11	7.8	16	6	0.067	0.032	0.1	10	6.9	22	63	61	65
5	f2ft5	50	2.67	11	4	20	1	130	80	200	4.2	3.1	5.3	1	0.049	0.025	0.077	14	9	28	28	24	31
6	LC20	50	2.67	54	31	80	8	640	430	820	15	11	19	8	0.071	0.041	0.11	9.8	6.3	17	70	68	72
7	LC14	50	2.67	34	16	55	4	410	220	600	10	6.2	14	3	0.066	0.04	0.095	11	7.3	17	62	60	64
8	T7	50	2.67	37	21	79	6	360	240	750	13	9.3	19	7	0.044	0.006	0.083	16	8.4	120	69	67	72
9	T2	50	2.67	27	3	60	3	260	83	490	10	7	15	4	0.012	-0.03	0.076	58	9.1		65	61	67
1	baseline	70	3.01	220	190	280	9	2600	2300	2800	59	48	66	9	0.094	0.067	0.12	7.4	5.8	10	89	88	90
2	CS25	70	3.01	140	98	180	5	1700	1400	1900	36	28	44	5	0.083	0.053	0.11	8.4	6.3	13	82	80	83
3	CS50	70	3.01	68	46	92	3	790	480	1100	18	11	23	2	0.071	0.043	0.11	9.8	6.3	16	70	68	71
4	f2ft15	70	3.01	160	120	200	7	1900	1500	2100	40	31	48	6	0.084	0.054	0.11	8.3	6.3	13	83	82	85
5	f2ft5	70	3.01	40	23	61	1	480	360	650	12	8.7	15	1	0.064	0.032	0.1	11	6.9	22	61	59	63
6	LC20	70	3.01	190	140	240	8	2300	1900	2500	50	39	57	8	0.089	0.058	0.12	7.8	5.8	12	87	86	88
7	LC14	70	3.01	140	88	170	6	1600	1200	1900	35	24	43	4	0.083	0.051	0.12	8.4	5.8	14	84	83	84
8	T7	70	3.01	130	88	160	4	1200	1000	1300	42	33	48	7	0.066	0.036	0.092	11	7.5	19	87	86	88
9	T2	70	3.01	58	30	180	2	500	320	1700	28	19	40	3	0.031	0.002	0.081	22	8.6	410	84	83	86
1	baseline	100	3.4	130	90	170	1	2000	1600	2500	94	92	94	9	0.12	0.098	0.15	5.8	4.6	7.1	97	96	97
2	CS25	100	3.4	210	150	250	7	2800	2300	3200	84	80	88	4	0.11	0.085	0.14	6.3	5	8.2	94	94	95
3	CS50	100	3.4	220	180	260	8	2700	2500	2900	62	51	70	2	0.094	0.069	0.12	7.4	5.8	10	88	87	89
4	f2ft15	100	3.4	200	140	260	6	2700	2100	3200	86	82	89	6	0.11	0.081	0.13	6.3	5.3	8.6	95	94	95
5	f2ft5	100	3.4	180	140	240	4	2200	1900	2500	47	36	57	1	0.087	0.034	0.12	8	5.8	20	83	82	84
6	LC20	100	3.4	160	120	220	3	2300	2000	3000	91	89	93	8	0.12	0.099	0.14	5.8	5	7	96	96	97
7	LC14	100	3.4	220	170	280	9	2900	2400	3300	85	81	88	5	0.11	0.078	0.13	6.3	5.3	8.9	95	94	95
8	T7	100	3.4	180	140	250	5	2000	1500	2400	87	83	89	7	0.096	0.07	0.12	7.2	5.8	9.9	96	96	97
9	T2	100	3.4	140	85	220	2	1200	740	2100	74	68	79	3	0.059	0.025	0.084	12	8.3	28	95	94	95

Supplementary table 1: Values of model outputs by intervention scenario and relative infectiousness of asymptomatic cases.

Supplementary References

1. Davies, N. G. *et al.* Age-dependent effects in the transmission and control of COVID-19 epidemics. *Nat. Med.* (2020) doi:10.1038/s41591-020-0962-9.
