# Supplementary material for: High COVID-19 transmission potential associated with re-opening universities can be mitigated with layered interventions
Source: Nat Commun. 2021 Aug 17;12:5017. doi: 10.1038/s41467-021-25169-3 (PMC8371131; doi:10.1038/s41467-021-25169-3)
Supplement: Supplementary file 3 — Reporting Summary [file 41467_2021_25169_MOESM3_ESM.pdf]

nature research

Corresponding author(s): Ellen Brooks-Pollock

Last updated by author(s): Jul 6, 2021

Reporting Summary

Nature Research wishes to improve the reproducibility of the work that we publish. This form provides structure for consistency and transparency in reporting. For further information on Nature Research policies, see our Editorial Policies and the Editorial Policy Checklist.

Statistics

For all statistical analyses, confirm that the following items are present in the figure legend, table legend, main text, or Methods section.

n/a	Confirmed	
☐	☒	The exact sample size (*n*) for each experimental group/condition, given as a discrete number and unit of measurement
☐	☒	A statement on whether measurements were taken from distinct samples or whether the same sample was measured repeatedly
☒	☐	The statistical test(s) used AND whether they are one- or two-sided<br>*Only common tests should be described solely by name; describe more complex techniques in the Methods section.*
☒	☐	A description of all covariates tested
☒	☐	A description of any assumptions or corrections, such as tests of normality and adjustment for multiple comparisons
☐	☒	A full description of the statistical parameters including central tendency (e.g. means) or other basic estimates (e.g. regression coefficient) AND variation (e.g. standard deviation) or associated estimates of uncertainty (e.g. confidence intervals)
☒	☐	For null hypothesis testing, the test statistic (e.g. *F*, *t*, *r*) with confidence intervals, effect sizes, degrees of freedom and *P* value noted<br>*Give P values as exact values whenever suitable.*
☒	☐	For Bayesian analysis, information on the choice of priors and Markov chain Monte Carlo settings
☒	☐	For hierarchical and complex designs, identification of the appropriate level for tests and full reporting of outcomes
☒	☐	Estimates of effect sizes (e.g. Cohen's *d*, Pearson's *r*), indicating how they were calculated

Our web collection on statistics for biologists contains articles on many of the points above.

Software and code

Policy information about availability of computer code

Data collection	No code was generated to collect data for this study.
Data analysis	The data were processed using R version 4.0.2 (2020-06-22). Model code for reproducing epidemic trajectories and other outputs is available at https://github.com/ellen-is/unimodel/ 35.

For manuscripts utilizing custom algorithms or software that are central to the research but not yet described in published literature, software must be made available to editors and reviewers. We strongly encourage code deposition in a community repository (e.g. GitHub). See the Nature Research guidelines for submitting code & software for further information.

Data

Policy information about availability of data

All manuscripts must include a data availability statement. This statement should provide the following information, where applicable:

- Accession codes, unique identifiers, or web links for publicly available datasets
- A list of figures that have associated raw data
- A description of any restrictions on data availability

The Social Contact Survey data used in this study are available at http://wrap.warwick.ac.uk/54273/). The raw UoB student data are protected and are not available due to data privacy laws. The aggregated UoB student contact matrices are available to download at https://github.com/ellen-is/unimodel/ (Brooks-Pollock, E. Stochastic transmission model of COVID-19 in a university setting v1.0.0. (2021) doi:10.5281/zenodo.4759117).

Field-specific reporting

Please select the one below that is the best fit for your research. If you are not sure, read the appropriate sections before making your selection.

☐ Life sciences ☒ Behavioural & social sciences ☐ Ecological, evolutionary & environmental sciences

For a reference copy of the document with all sections, see nature.com/documents/nr-reporting-summary-flat.pdf

Behavioural & social sciences study design

All studies must disclose on these points even when the disclosure is negative.

Study description	Mathematical modelling study
Research sample	All students registered at the University of Bristol on 1 October 2019
Sampling strategy	All students registered in the 2019/2020 academic year. As all registered students are included no sample size calculations were necessary.
Data collection	The study used re-purposed data, therefore no data were collected for this study. The data were collected using a standardised registration procedure, administered by the central University of Bristol administration team.
Timing	start date for the 2019/2020 academic year: 23 September 2019; end date: 12 June 2020
Data exclusions	No records were excluded.
Non-participation	We were provided with an anonymised extract of student data for a university relating to the 2019/2020 academic year. The study complied with the University data protection policy for research studies (http://www.bristol.ac.uk/media-library/sites/secretary/documents/information-governance/data-protection-policy.pdf).
Randomization	No randomisation was used, as we used data relating to all UoB students. We used the data for descriptive data analysis, therefore no covariates were considered.

Reporting for specific materials, systems and methods

We require information from authors about some types of materials, experimental systems and methods used in many studies. Here, indicate whether each material, system or method listed is relevant to your study. If you are not sure if a list item applies to your research, read the appropriate section before selecting a response.

Materials & experimental systems

n/a	Involved in the study
☒	☐ Antibodies
☒	☐ Eukaryotic cell lines
☒	☐ Palaeontology and archaeology
☒	☐ Animals and other organisms
☒	☐ Human research participants
☒	☐ Clinical data
☒	☐ Dual use research of concern

Methods

n/a	Involved in the study
☒	☐ ChIP-seq
☒	☐ Flow cytometry
☒	☐ MRI-based neuroimaging
